# Lipid reprogramming induced by the TFEB-ERRα axis enhanced membrane fluidity to promote EC progression

**DOI:** 10.1186/s13046-021-02211-2

**Published:** 2022-01-19

**Authors:** Xiaodan Mao, Huifang Lei, Tianjin Yi, Pingping Su, Shuting Tang, Yao Tong, Binhua Dong, Guanyu Ruan, Alexander Mustea, Jalid Sehouli, Pengming Sun

**Affiliations:** 1 Laboratory of Gynecologic Oncology, Department of Gynecology, Fujian Maternity and Child Health Hospital, Affiliated Hospital of Fujian Medical University. No, 18 Daoshan Road, Fuzhou, 350001 China; 2 School of Medical Technology and Engineering, Fujian Medical University, No.88 Jiaotong Road, Fuzhou, 350004 China; 3 Fujian Key Laboratory of Women and Children’s Critical Diseases Research, No. 18 Daoshan Road, Fuzhou, 350001 China; 4 National Health Commission Key Laboratory of Technical Evaluation of Fertility Regulation for Non-Human Primate, Jinjishan Road 19, Fuzhou, 350005 China; 5 Department of Obstetrics and Gynecology, Key Laboratory of Birth Defects and Related Diseases of Women and Children of MOE and State Key Laboratory of Biotherapy, West China Second University Hospital, Sichuan University and Collaborative Innovation Center, Chengdu, 610041 China; 6 Department of Gynecology and Gynecological Oncology, University Hospital Bonn, Venusberg-Campus 1, 53127 Bonn, Germany; 7 Department of Gynecology and Obstetrics, Charité Virchow University Hospital, Augustenberger Platz 1, 13353 Berlin, Germany

**Keywords:** TFEB, ERRα, Endometrial cancer, Lipid reprogramming, Mitochondrial stress, EMT signaling

## Abstract

**Background:**

Estrogen-related receptor α (ERRα) has been reported to play a critical role in endometrial cancer (EC) progression. However, the underlying mechanism of ERRα-mediated lipid reprogramming in EC remains elusive. The transcription factor EB (TFEB)-ERRα axis induces lipid reprogramming to promote progression of EC was explored in this study.

**Methods:**

TFEB and ERRα were analyzed and validated by RNA-sequencing data from the Cancer Genome Atlas (TCGA). The TFEB-ERRα axis was assessed by dual-luciferase reporter and chromatin immunoprecipitation quantitative polymerase chain reaction (ChIP-qPCR). The mechanism was investigated using loss-of-function and gain-of-function assays in vitro. Lipidomics and proteomics were performed to identify the TFEB-ERRα-related lipid metabolism pathway. Pseudopods were observed by scanning electron microscope. Furthermore, immunohistochemistry and lipidomics were performed in clinical tissue samples to validate the ERRα-related lipids.

**Results:**

TFEB and ERRα were highly expressed in EC patients and correlated to EC progression. ERRα is the direct target of TFEB to mediate EC lipid metabolism. TFEB-ERRα axis mainly affected glycerophospholipids (GPs) and significantly elevated the ratio of phosphatidylcholine (PC)/sphingomyelin (SM), which indicated the enhanced membrane fluidity. TFEB-ERRα axis induced the mitochondria specific phosphatidylglycerol (PG) (18:1/22:6) + H increasing. The lipid reprogramming was mainly related to mitochondrial function though combining lipidomics and proteomics. The maximum oxygen consumption rate (OCR), ATP and lipid-related genes *acc, fasn,* and *acadm* were found to be positively correlated with TFEB/ERRα. TFEB-ERRα axis enhanced generation of pseudopodia to increase the invasiveness. Mechanistically, our functional assays indicated that TFEB promoted EC cell migration in an ERRα-dependent manner via EMT signaling. Consistent with the in vitro, higher PC (18:1/18:2) + HCOO was found in EC patients, and those with higher TFEB/ERRα had deeper myometrial invasion and lower serum HDL levels. Importantly, PC (18:1/18:2) + HCOO was an independent risk factor positively related to ERRα for lymph node metastasis.

**Conclusion:**

Lipid reprogramming induced by the TFEB-ERRα axis increases unsaturated fatty acid (UFA)-containing PCs, PG, PC/SM and pseudopodia, which enhance membrane fluidity via EMT signaling to promote EC progression. PG (18:1/22:6) + H induced by TFEB-ERRα axis was involved in tumorigenesis and PC (18:1/18:2) + HCOO was the ERRα-dependent lipid to mediate EC metastasis.

**Supplementary Information:**

The online version contains supplementary material available at 10.1186/s13046-021-02211-2.

## Background

High body mass index (BMI) influences the current and future health of patients and is considered one of the top 5 global causes of death for females [1]. Among all female malignancies, endometrial cancer (EC) is most strongly associated with obesity, and obesity has been considered a very important risk factor for EC in postmenopausal women [2]. EC is the sixth most common cancer in women and the second most common gynecological malignancy globally. There were approximately 66,570 new cases and 12,940 reported deaths due to EC in the United States in 2021 [3]. The incidence of EC has also markedly increased in China recently. The age-standardized incidence of EC was 63.4 per 100,000, the mortality rate was 21.8 per 100,000, and the 5-year relative survival was 72.8% from 2012–2015 [4]. Considering the accompanying symptoms of overweight and diabetes in EC patients, tumor lipid metabolism has been the research focus. However, the mechanism of lipid metabolism in EC is still unclear.

Estrogen-related receptor α (ERRα, NR3B1, ESRRA) is a constitutively active ligand-independent orphan nuclear receptor that belongs to the nuclear receptor superfamily [5]. As a transcription factor, ERRα combines with its acknowledged coactivator peroxisome proliferator-activated receptor (PPAR) coactivator-1α (PGC-1α) and plays a central role in the regulation of cellular oxidative phosphorylation and liposome metabolism, resulting in many biological functions [6, 7]. Consistent with an increasing number of studies on breast cancer [8], colon cancer [9] and ovarian cancer [10], our previous works confirmed that high expression of ERRα was significantly related to a poor prognosis in EC [10]. Moreover, overexpression of ERRα can downregulate the expression of E-cadherin while upregulating the expression of vimentin and inducing epithelial-mesenchymal transformation (EMT), which promotes invasion and migration, indicating that it may be a new biomarker for predicting the risk of deep myometrial invasion and metastasis [11]. After targeted inhibition of ERRα by small interfering RNA (siRNA) or antagonist XCT790, transcription factor EB (TFEB) was identified as a potential interacting protein by a DNA/protein high-throughput assay [12]. TFEB, a master regulator of lysosomal biogenesis and autophagy, was found to have a crucial pathogenic role in different tumors [13]. Several recent studies have also focused on the function of TFEB in tumor cell metabolism. Carmine et al. suggested that TFEB might be a novel therapeutic target for disorders of lipid metabolism, such as fatty liver disease, and that TFEB exerts global transcriptional control on lipid catabolism via PGC-1α and PPARα [14]. However, there were only very few reports about the association between TFEB and ERRα aside from those published by our team.

The EMT program is related to lipid remodeling of the cell membrane [15]. The fatty acyl moieties of membrane phospholipids exhibit considerable diversity in chain length and different degrees of saturation, which determine the biophysical properties of membranes, including their fluidity, curvature, and subdomain architecture [16]. The major structural phospholipids in mammalian membranes are glycerophospholipids (GPs), among which phosphatidylcholine (PC) is the most abundant in mammalian cell membranes and subcellular organelles, accounting for 40–50% of total phospholipids [17]. The saturability of PC affects the plasma membrane of tumor cells to sustain oncogenic activity in a wide variety of cancers [16, 18]. Lin et al. showed that the length of the fatty acid chain in the membrane modulated plasma membrane fluidity and invasion of liver cancer [19]. The roles of lipid metabolism in the function of the membrane in EC still need to be unveiled. Interestingly, both ERRα and TFEB were reported to be involved in the lipid remodeling signaling pathway.

How ERRα and TFEB play roles in lipid metabolism and how this mechanism affects malignant cell metastasis and invasion still need more research. We hypothesized that TFEB-ERRα signaling, which regulates lipid metabolism, extensively affects membrane function to promote EC invasion and metastasis. In this work, our discovery discusses the crosstalk between TFEB and ERRα and their coregulation of FA metabolism to promote invasion and metastasis in EC.

## Materials and methods

### Cell lines and cell culture

Human KLE endometrial adenocarcinoma cells were obtained from the Shanghai Cell Biological Research Institute (Shanghai, China), and ECC-1 cells were acquired from the American Type Culture Collection (ATCC, USA). KLE cells are ERα-, while ECC-1 cells are ERα + . KLE and ECC-1 cells were thawed and cultured in DMEM/F12 medium (#A4192001, Thermo Fisher, Waltham, MA, USA), 1% antibiotic–antimycotic solution (#B120901, BasalMedia, Shanghai, China), and 10% fetal bovine serum (FBS) (#2275129, Gibco,  Inchinnan, UK) at 37 °C in 5% CO_2_. Cells treated with XCT790 (#725247-18-7, Sigma-Aldrich, St. Louis, MO, USA) were incubated in phenol red-free medium (#21041025, Thermo Fisher, Waltham, MA, USA) containing 1% serum replacement 1 (#S0638, Sigma-Aldrich, St. Louis, MO, USA). ECC-1 and KLE were incubated with 10 µM XCT790 (in dimethyl sulfoxide [DMSO]; #Y190601, MP Biomedicals LLC, Santa Ana, CA, USA) or DMSO (control) for 24 h. Lentiviral vectors expressing siRNAs targeting TFEB (named TFEB-KD) and ERRα (named ERRα-KD) were constructed. The following siRNA target sequence in the TFEB gene (GenBank accession No. NM_001167827.3) was selected: 5′-GAG ACG AAG GTT CAA CAT CAA-3'. The siRNA target sequence in the ERRα (GenBank accession NM_004451.5) was 5'-GAG CGA GAG TAT GTT CTA-3′. The lentiviral vector used to overexpress TFEB and ERRα were named TFEB-OV and ERRα-OV, respectively. Overexpression of ERRα or TFEB was achieved in KLE and ECC-1 cells and named KLE^TFEB−OV^, ECC-1^TFEB−OV^, KLE^ERRα−OV^ and ECC-1^ERRα−OV^, respectively. In addition, KLE and ECC-1 cells with ERRα or TFEB expression downregulated through lentivirus-mediated siRNA were named KLE^TFEB−KD^, ECC-1^TFEB−KD^, KLE^ERRα−KD^ and ECC-1^ERRα−KD^, respectively.

### Bioinformatics data analyze

The Cancer Genome Atlas (TCGA) data of Uterine Corpus Endometrial Carcinoma (UCEC) were downloaded from Genomic Data Commons data portal https://portal.gdc.cancer.gov/. The dataset included 23 normal endometrial specimens and 543 EC specimens, with 9 repeated cancerous specimens excluded. Patient clinical information, gene-level copy number variation (CNV) profiles, gistic2 thresholds analyzed by the GISTIC2.0 method and somatic nonsilent mutation (gene-level) data were acquired from the University of California, Santa Cruz (UCSC) Xena website. The Database for Annotation, Visualization and Integrated Discovery (DAVID) (version 6.8) provides a comprehensive set of functional annotation tools that help investigators understand the biological meaning behind a large list of genes. GO functional annotation and KEGG analysis of the proteins were performed, and the results were visualized with the cluster Profiler R package (version 1.3.1093).

### RNA extraction, RT-qPCR, Western blotting (WB)

Samples were collected from an equal number of intact cells in TRI Reagent® (#TR118; Molecular Research Center, Cincinnati, OH, USA). After reverse transcription on 500 ng of total RNA with GoScript^TM^ Reverse Transcription Mix, Oligo (dT) (#A2791, Promega, Madison, Wis, USA), quantitative PCR amplification was performed on the LightCycler 480 (Roche, Switzerland) using Eastep® qPCR Master Mix Kit (#LS2062, Promega, Madison, Wis, USA). Relative gene expression was calculated using the 2^−ΔΔCt^ method, with GAPDH as the reference gene. Standard techniques were used for protein quantification, separation, transfer, and blotting in ECC-1 and KLE cells. Primary antibodies against the following targets were used: TFEB (1:1000; #ab270604, Abcam, London, UK), ERRα (1:500; #ab137489, Abcam, London, UK), LPCAT1 (1:1000; #66044-1-Ig, Proteintech, Wuhan, China), LPCAT3 (1:1000; #67882-1-Ig, Proteintech, Wuhan, China), MMP2 (1:1000; #AF1420, Beyotime Biotechnology, Shanghai, China), Cortactin (1:1000; #AF2134, Beyotime Biotechnology, Shanghai, China), E-cadherin (1:1000; #3195, Cell Signaling Technology, Shanghai, China), vimentin (1:1000; #5741, Cell Signaling Technology, Shanghai, China), and GAPDH (1:2000; #60004-1-Ig, Proteintech, Wuhan, China).

### Wound Healing

Cells were grown to confluence in 6-well plates, and a 200-µL tip was used to introduce a scratch in the monolayer. The scratch areas in the wells were washed with PBS and 1 mmol/L R-flurbiprofen until the cells in those areas were removed thoroughly and imaged at 0 and 24 h post-scratching. The horizontal migration rate was calculated using the following formula: (width 0 h − width 24 h)/width 0 h × 100%.

### Chromatin-Immunoprecipitation (ChIP) assay

Cells were harvested followed by cross-linking for 10 min with 1% (v/v) formaldehyde. Afterward, cells were lysed by sonication. The cell lysates were immunoprecipitated with anti-TFEB (1:100; ab270604, Abcam, London, UK) overnight at 4 °C. After washing and elution, the crosslinks were reversed for 4 h at 65 °C. The eluted DNA was purified and analyzed by qPCR using a Bio-Rad SYBR Green intercalating fluorophore system with the following ERRα primers: 5’-AGT TGT GAG GAG CCT TTG GAC-3’ (forward) and 5’-CGG TGG TAG CGA GCA GTT T-3’ (reverse). The Ct value of each sample was normalized to the corresponding input value.

### Luciferase reporter assays

Bioinformatics methods were used to analyze and predict the potential transcription factor binding sites in the ERRα promoter region. The ERRα promoter sequence (64,303,524 bp to 64,305,524 bp) relative to the transcription start site was amplified by PCR and inserted into the pGL3-basic vector (#E1751, Promega, Madison, Wis, USA). KLE cells were cotransfected with empty pcDNA3.1 vector or TFEB-S211A pcDNA3.1 plasmid in 24-well plates with Lipofectamine 2000. After 48 h, the firefly and Renilla luciferase activities were measured using the Dual-Luciferase Reporter Assay Kit (#E1901, Promega, Madison, Wis, USA) and a microplate reader (Synergy H1, Bio-Tek, USA), and the ratio of firefly/Renilla luciferase activity was determined.

### Lipid and metabolite profiling

Liquid chromatography mass spectrometry (LC/MS) analyses were performed using a high-performance liquid chromatography system (1260 series; Agilent Technologies, USA) and mass spectrometer (Agilent 6460; Agilent Technologies, USA). Briefly, 10^7^/ml EC cells or 20 mg EC tissue was homogenized in 1.5 mL of chloroform/methanol (2:1, v/v), vortexed for 1 min, centrifuged at 3,000 rpm for 10 min, added to 800 µL organic phase in a clean tube, and dried with nitrogen. Sample preparation processes were performed in accordance with the above method of parallel preparation of quality control samples. Mass spectrometric analysis was conducted by adding 200 µL isopropanol/methanol solution (1:1, v:v), and the supernatant was used for analysis. For targeted metabolomic analyses, multiple reaction monitoring transitions representing the metabolites were simultaneously monitored, and positive/negative polarity switching was used. Data analyses were performed according to the instructions of Shanghai Applied Protein Technology [20].

### Tandem mass tag (TMT) labeling proteomics

The total protein in KLE cells and KLE^XCT790^ cells dealed with 10 μM XCT790 were extracted and evaluated by SDS-PAGE and staining. The qualified protein samples were labeled with trypsin and TMT. The labeled polypeptides were mixed into one component in equal quantities. After desalination, high-pH HPLC was used for grading. Eight different polypeptide components were obtained, and each component was separated by nano-HPLC and detected by mass spectrometry. Then, maxQuant search software was used for protein identification and quantitative analysis. After the quantitative results were standardized, statistical analysis was conducted to screen out the differentially expressed proteins.

### Mitochondrial stress detection

Mitochondrial stress detection was conducted by using the Seahorse XF Cell Mitochondrial Stress Test Kit (#103,015–100, Agilent, USA). In brief, the test solution based on DMEM medium (#103,575–100, Agilent, USA) was heated in 37 °C and working solution was prepared for use. Oligomycin, 0.5 µM FCCP, and rotenone/antimycin A were properly prepared into a working solution and added to the dole on the probe plate. Cell culture microplates were removed from a 37 °C CO_2_ incubator, and the cells were examined under a microscope to confirm the 90% of confluence. Remove the test solution from the water bath. The cell growth medium in the cell culture microplates was replaced with preheated detection solution using a multichannel pipette, and the cell culture microplates were placed in a CO_2_-free incubator at 37 °C for 1 h. Then, run the Seahorse XF 24 (Agilent, USA) on the computer and analyze the data.

### Immunohistochemistry (IHC)

All tissues were assembled into a tissue microarray. Immunostaining for ERRα and TFEB was performed according to standard procedures. Rabbit polyclonal anti-ERRα (1:100; #ab137489, Abcam, London, UK) and rabbit polyclonal anti-TFEB (1:100; #ab270604, Abcam, London, UK) antibodies were used. The percentage of positive cells was scored as 0 (cells < 5%), 1 (5% to 25%), 2 (26% to 50%), 3 (51% to 75%), and 4 (76% to 100%). The positive staining intensity was scored as 0 (no staining), 1 (weak staining), 2 (moderate staining), and 3 (strong staining). The expression levels of ERRα and TFEB were assessed to determine their immunoreactive scores (IRSs) using the algorithm IRS = Si × Pi (where Si and Pi represent the intensity and percentage of positively stained cells, respectively). Samples were divided into four groups based on their IRS: 0, negative (-); 1–4, weakly positive ( +); 5–8, positive (+ +); and 9–12, strongly positive (+ + +).

### Scanning Electron Microscope (SEM)

Briefly, cells were cultured on petri dish and treated with electron microscopy fixative (#G1102, Servicebio, China). After post-fixing, dehydrate cells with 30%, 50%, 70%, 80%, 90%, 95% ethanol (#100,092,183, Sinaopharm Group Chemical Reagent Co. LTD, China) for 15 min, respectively. Dry samples with Critical Point Dryer. Specimens are attached to metallic stubs using carbon stickers and sputter-coated with gold for 30 s. Micrographs were revealed using HITACHI-SU8100 scanning electron microscope.

### Participants and specimens

EC tissue and normal endometrial tissue samples and blood samples with relevant clinical data were obtained from surgical patients in Fujian Provincial Maternity and Child Health Hospital, Affiliated Hospital of Fujian Medical University from 2013 to 2018. None of the patients received any preoperative radiation, chemotherapy or hormone therapy. Finally, we collected 111 tissue specimens, including 79 EC specimens and 32 normal endometrium specimens. The samples were embedded in paraffin, and all diagnoses of the pathological sections were made by experienced pathologists. In addition, according to ERRα immunoreactive scores, 35 cases with the highest score and 20 cases with the lowest score were selected for lipidomic analysis. Finally, because 1 EC tissue sample was missed (IRS = 7). A total of 51 patient tissues from 32 EC patients and 19 patients with normal endometrium were also collected for lipidomics analysis 3 samples were excluded because of metabolite degradation (Supplement Figure 1). All patients were informed of the experiments and signed informed consent forms. This research protocol was approved by the Ethics Committee of Fujian Provincial Maternity and Child Health Hospital, Affiliated Hospital of Fujian Medical University (No. FMCH-2018–14).

**Fig. 1:**
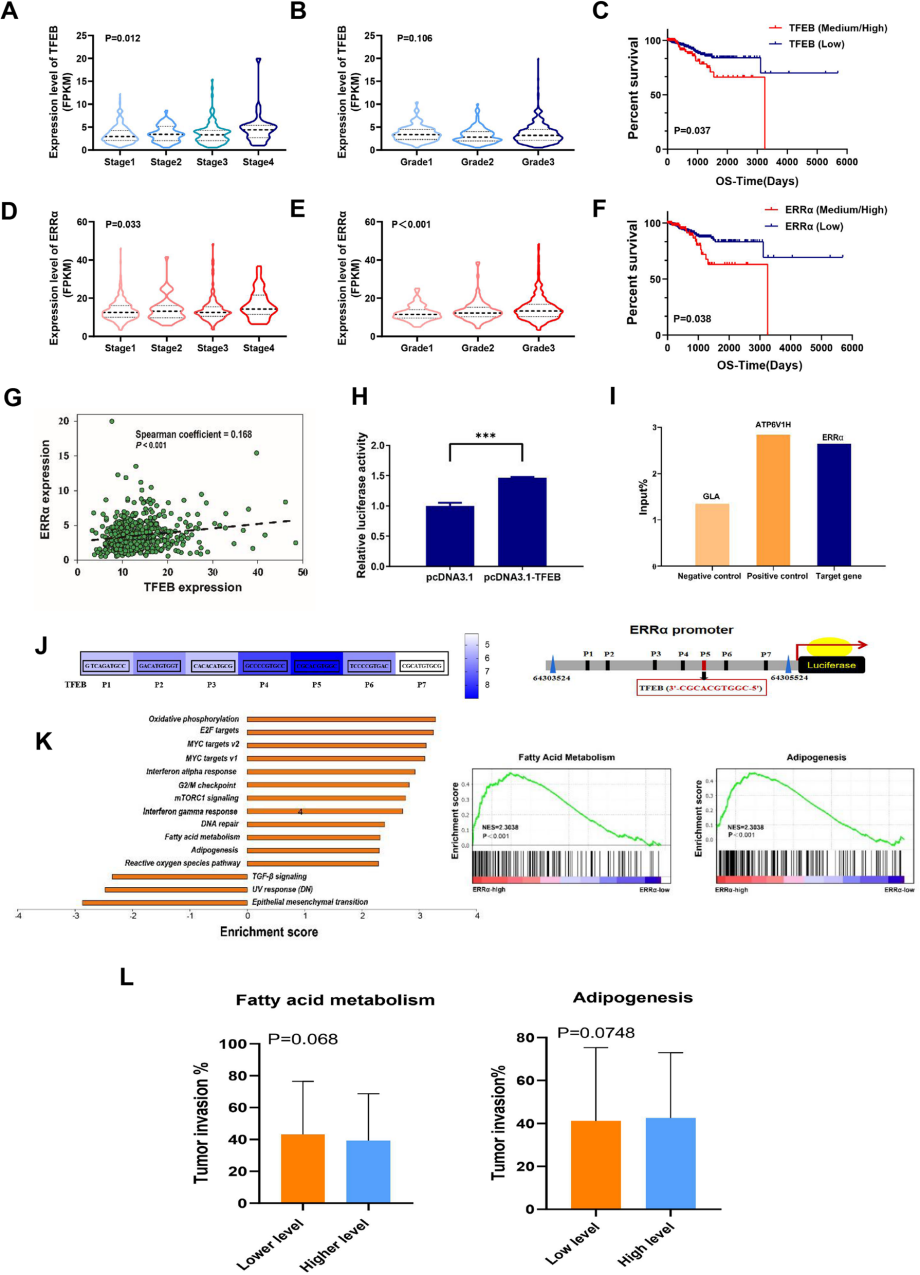
Bioinformatics analysis revealed that TFEB promotes ERRα transcription to participate in EC progression TCGA database (Sample size: Normal = 23; EC = 543) results are shown. (**A)** The expression of TFEB varies at different FIGO stages (**B)** and at different pathological grades. (**C)** The association of TFEB with OS in the patient/specimen quartiles is shown (Low: 1st quartile distribution; Median: 2nd-3rd quartile distribution; High: 4th quartile distribution). (**D)** The expression of ERRα varies at different FIGO stages (**E)** and at different pathological grades. (**F)** The association of ERRα with OS is shown. (**G)** The correlation between the expression levels of TFEB and ERRα in EC tissue. **(H**) ChIP analysis of the ERRα promoter occupancy in KLE cells is performed as described in the Materials and Methods section. TFEB is immunoprecipitated using an anti-Flag antibody, and DNA enrichment is performed using qPCR. The ATP6V1H promoter is used as a positive control and the GLA promoter is used as a negative control. (**I)** KLE cells are co-transfected with Flag-TFEB, ERRα promoter labeled with luciferase reporter, and Renilla luciferase control. 48 h after transfection, the cells are analyzed and the relative luciferase activity is measured and normalized to the Renilla luciferase control. (**J)** The putative ERRα-binding sites (ERREs), as predicted by the online program JASPAR (https://jaspar.genereg.net/analysis), are located in the TFEB (P1-P7) gene promoter regulatory regions. (**K**) KEGG pathway analysis (Ordinate: the KEGG signal path; abscissa: enrichment score). Results of GSEA in fatty acid metabolism and adipogenesis pathways. (**L**) The association of fatty acid metabolism and adipogenesis with tumor invasion is shown (Low: 1st- 2nd quartile distribution; High: 3rd-4th quartile distribution). Statistical tests: ANOVA (A-B, D-E), Kaplan–Meier estimator (C, F), Pearson correlation analysis (G), and Student’ s *t*-test (L). p < 0.05 suggests significantly different. TCGA: The Cancer Genome Atlas; FIGO: Federation International of Gynecology and Obstetrics; OS: Overall Survival; ChIP: Chromatin Immunoprecipitation; KEGG: Kyoto Encyclopaedia of Genes and Genomes; GSEA: Gene Set Enrichment Analysis.

### Statistical analysis

Statistical analysis was performed using GraphPad Prism 8.0 software and IBM SPSS (version 22). Statistical significance was determined by Student’s *t* test or by ANOVA, and related parameters were analyzed using Pearson’s correlation. Correlation coefficients for graded data were obtained using Pearson correlation analysis. Receiver operating characteristic (ROC) curves and the Youden Index were used to determine the cutoff point of continuous variables. Univariate binary logistic regression analyses were used to analyze indicators associated with EC. Differences with p-values less than 0.05 were considered significant.

## Results

### Bioinformatics analysis revealed that TFEB promotes ERRα transcription to participate in EC progression

To explore the role of TFEB and ERRα in EC, we first investigated the expression and clinicopathological data of these two genes in 543 EC samples and 23 normal samples from TCGA RNA-seq database. High expression of TFEB was significantly associated with a more advanced stage (p <0.05; Fig. 1A) but not with pathological grade in EC (p >0.05; Fig. 1B). Moreover, EC patients with high expression of TFEB had worse overall survival (OS) than those with low expression (p <0.05; Fig. 1C). Similarly, high ERRα expression was significantly associated not only with more advanced stages but also grades in EC (both p <0.05; Fig. 1D-F). Consistent with our purpose, the expression of TFEB was significantly positively correlated with ERRα (Pearson coefficient = 0.168; p <0.001; Fig. 1G).

### ERRα is a direct transcriptional target of TFEB involved in lipid metabolism in EC

Previously, we demonstrated that the transcriptional activity of TFEB correlated with ERRα, but the exact mechanism remains unclear. Luciferase activity detection showed that the relative luciferase activity triggered by ERRα expression was significantly enhanced by the promotion of TFEB (Fig. 1H). To further study the crosstalk between TFEB and ERRα, ChIP-qPCR was performed, and the results confirmed that TFEB could directly bind to the promoter of ERRα DNA (Fig. 1I). Seven possible TFEB transcriptional binding sites (Fig. 1J; region P1-P7; all relative scores > 0.80) on the promoter region of the ERRα gene were predicted. Among them, the P5 site with the element sequence 3’-CGCACGTGGC-5’ was the most likely combination with TFEB (Fig. 1J). These data strongly indicate that TFEB could directly bind to the ERRα promoter and positively regulate ERRα expression. ERRα is the key regulator involved in lipid catabolism. Gene set enrichment analysis (GSEA) of the high- and low-ERRα expression groups was conducted to explore and identify the potential function of ERRα in EC. The gene sets with nominal p < 0.05 and false discovery rate (FDR) < 0.25 were considered significantly enriched in fatty acid (FA) metabolism and adipogenesis (Fig. 1K). Then, we found FA metabolism and adipogenesis pathway both were nearly significantly associated with tumor invasion (p = 0.068 and p = 0.075, respectively, Fig. 1L), which suggested that lipid metabolism might play an important role in the invasion of EC.

### ERRα elevated unsaturated fatty acid (UFA)-containing GPs in EC

**Fig. 2:**
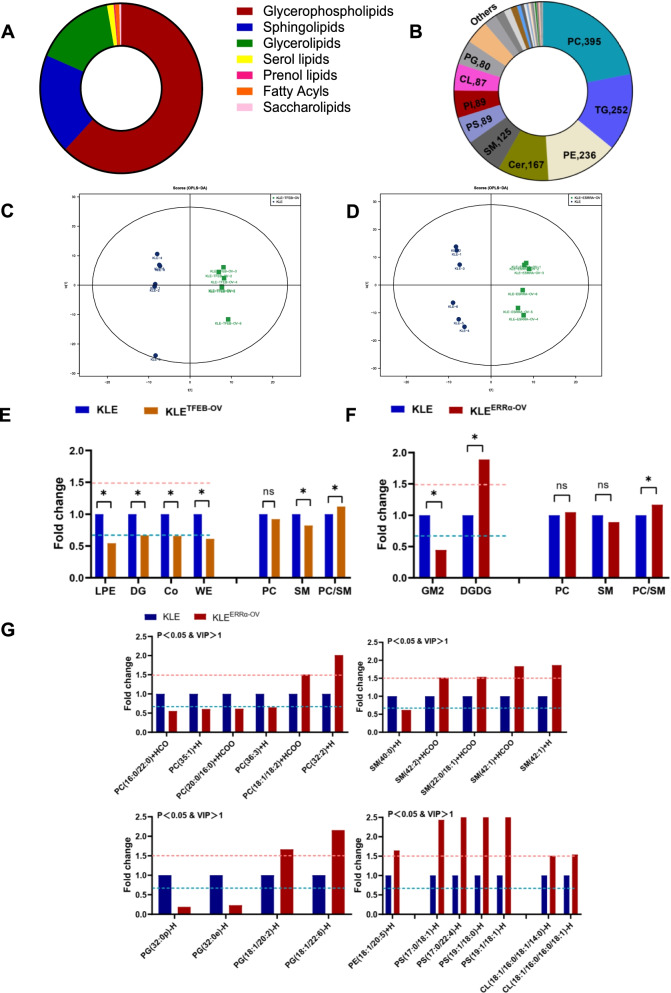
ERRα elevated unsaturated fatty acid (UFA)-containing GPs in EC (**A**) Seven subgroups of lipids in KLE cells are detected using lipidomics. (**B)** 36 classes of lipids were tested in KLE cells. (**C)** Systematic lipidomic changes between KLE and KLE^TFEB−OV^ assessed by orthogonal partial least squares-discriminant analysis (OPLS-DA). (**D**) Systematic lipidomic changes between KLE and KLE^ERRα−OV^ assessed by OPLS-DA. (**E**) Significant lipid species associated with TFEB and its PC/SM evaluation. (**F-G**) Significant lipid species associated with ERRα and its PC/SM evaluation. (p < 0.05 and VIP > 1 indicate significant). *, p < 0.05. Statistical tests: Student’ s *t*-test. TFEB-OV: TFEB Over-Expressed; ERRα-OV: ERRα Over-Expressed.

Subsequently, lipidomics was performed and analyzed in KLE^TFEB−OV^ cells and KLE^ERRα−OV^ cells. In general, 7 categories of lipids, which were composed of 1120 glycerophospholipids (GPs), 345 sphingolipids (SPs), 285 glycerolipids (GLs) and other lipid categories, were screened and identified based on a LC–MS/MS system (Fig. 2A). Finally, 36 classes of lipids were tested, which included 395 phosphatidylcholines (PCs), 252 triacylglycerols (TAGs), 236 phosphatidylethanolamines (PEs) and other lipid species (Fig. 2B). Systematic lipidomic changes occurring between KLE^TFEB−OV^ and KLE^ERRα−OV^ were then assessed by orthogonal partial least squares-discriminant analysis (OPLS-DA). There was obvious heterogeneity both in KLE^TFEB−OV^ and KLE^ERRα−OV^ with R^2^Y = 0.994, Q^2^ = 0.910 and R^2^Y = 0.989, Q^2^ = 0.838, respectively (Fig. 2C-D). Lysophosphatidylethanolamine (LPE), diglyceride (DG), coenzyme (Co) and wax exters (WE) was observed to decrease remarkably in KLE^TFEB−OV^. And digalactosyldiacylglycerol (DGDG) was observed to increase statistically, while gangliosides2 (GM2) was reduced significantly in KLE^ERRα−OV^. Although PC and sphingomyelin (SM) changed with TFEB or ERRα elevation indistinctively, the ratio of PC/SM was distinctly increased both in KLE^TFEB−OV^ and KLE^ERRα−OV^, which was used to evaluate cell membrane fluidity (p < 0.05, Fig. 2E-F).

As the direct target of TFEB, ERRα obviously elevates 7 species of lipids, including PCs, phosphatidylglycerols (PGs), cardiolipins (CLs), PE (18:1/20:5), phosphatidylserines (PSs) and ceramides (Cers). Increased UFA-containing PCs, PGs, PSs, SMs, CLs and PE (18:1/20:5) and a decrease in saturated fatty acid (SFA)-containing PCs, PGs and SM (40:0) were also detected in KLE^ERR−OV^ (Fig. 2G). Moreover, the overlapping lipids of KLE^TFEB−OV^ and KLE^ERRα−OV^ were PC (35:1) + H, PC (36:3) + H and PG (18:1/22:6) + H, respectively (Supplement Table 1). In brief, the common event is that UFA-containing GPs are increased in ERRα-overexpressing EC cells. The data showed that the membrane fluidity of KLE was increased with ERRα. Hence, TFEB drives ERRα to elevate the unsaturation of fatty acyl moieties in GPs, which enhances membrane fluidity for invasion and metastasis.

### Proteins/lipids related to ERRα were enriched in mitochondrial function in EC

**Fig. 3:**
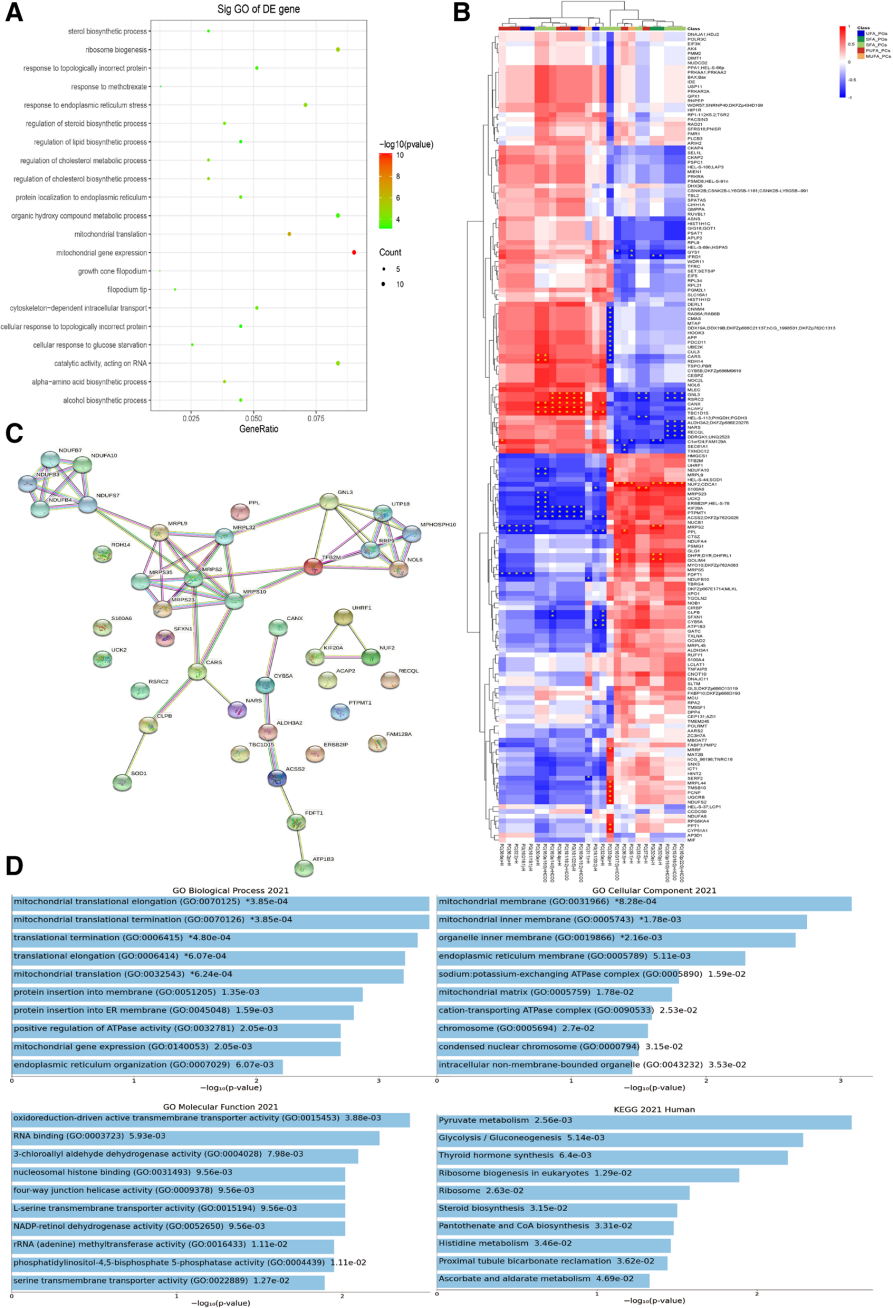
Proteins/lipids related to ERRα were enriched mitochondrial function of EC (**A**) Enrichment analysis for canonical pathways (CP) and biofunctions (BF) was performed on proteins related to ERRα. (**B**) 173 proteins related to ERRα were gained in KLE cells treatment with 10 μM XCT790 for 24 h using proteomics. (Blue, down-regulated; Red, up-regulated) (**C**)The significant protein related to the degree of unsaturated lipids were analyzed by STRING. (**D**) Mitochondrial function and glucolipid metabolism were enriched by GO and KEGG.

**Fig. 4:**
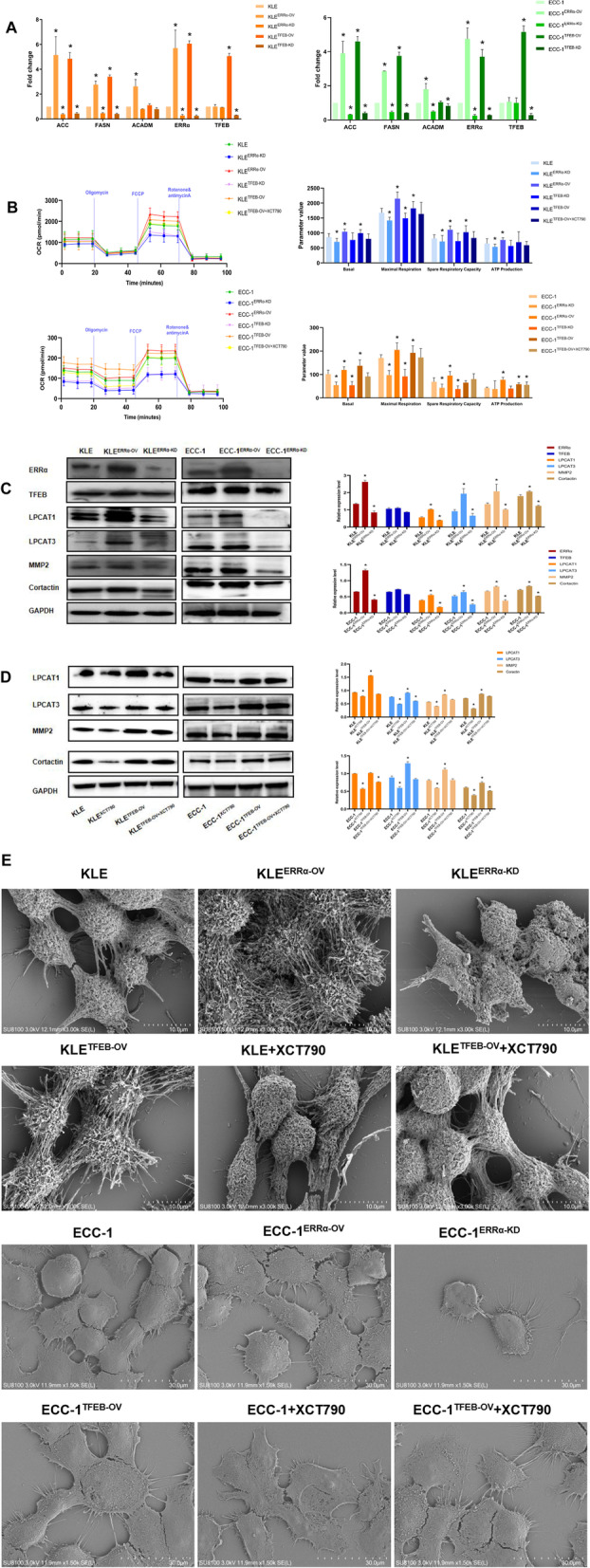
Proteins/lipids related to ERRα were enriched in mitochondrial function in EC (**A**) The relationship between TFEB-ERRα and *acc, fasn, acadm* are determined by RT-qPCR in KLE and ECC-1 cells. (**B**) The association of oxygen consumption rates (OCR), basal, maximal respiration, spare respiratory capacity, and ATP production regulated by TFEB-ERRα are shown. (**C**) The effect of ERRα regulation on TFEB, LPCAT1, LPCAT3, MMP2, and Cortactin expression in EC cells is analyzed using western blot (WB). (**D**) The effect of XCT790 treatment on LPCAT1, LPCAT3, MMP2, and Cortactin proteins for 24 h were evaluated between control and TFEB-overexpressing by WB. (**E**) Representative Scanning electron microscope (SEM) micrographs of KLE and ECC-1: TFEB-ERRα axis and XCT790 treatment for 24 h regulated pseudopod, in comparison to the controlled group. Micrographs are screened at scale of 10–30 µM. *, p < 0.05. Statistical tests: Student’ s *t*-test or ANOVA.

Compared to control KLE cells, 173 proteins related to ERRα with unique peptides ≥ 2.0, FC > 1.3 and p < 1.0 were gained in KLE cells treated with XCT790. The biological process of these proteins was mainly enriched in mitochondrial function, and the cell component was enriched in cone filopodium growth (Fig. 3A). Since an identified potential biological process was found to be affected by ERRα, the concentrations of these proteins/lipids were next evaluated in EC invasion and metastasis. Thirty-two proteins were significantly different between UFA-containing PCs, PGs, SMs and SFA-containing PCs, PGs, and SMs by combining proteomics and lipidomics (Fig. 3B-C, Supplement Table 2). The mitochondrial function was mainly enriched by STRING analysis (Fig. 3D). Then, we routinely detected genes associated with FA metabolism, including *acc*, *fasn* and *acadm*, which showed that FA metabolism was dynamic. Generally, all the gens were upregulated as TFEB-ERRα increased and downregulated as TFEB-ERRα decreased in KLE and ECC-1 cells (Fig. 4A). Subsequently, mitochondrial stress was evaluated by an energy analyzer. Compared to that of their controls, the maximum oxygen consumption rate (OCR) of cells treated with 0.5 µM FCCP was increased in KLE^TFEB−OV^ and KLE^ERRα−OV^, which was decreased in KLE^TFEB−KD^ and KLE^ERRα−KD^ cells (p < 0.05). The OCR related indicators, including basal, maximal respiration, and spare respiratory capacity were significantly higher in KLE^TFEB−OV^ and KLE^ERRα−OV^ cells (p < 0.05). Meanwhile, in KLE^TFEB−KD^ and KLE^ERRα−KD^ cells, the decreasing level of basal, maximal respiration, and spare respiratory capacity was also showed (p < 0.05). Moreover, the level of ATP also showed a positive correlation of TFEB and ERRα (p < 0.05). A similar trend was observed in ECC-1 cells. To further confirm the effects of TFEB on ERRα-mediated mitochondrial stress, ERRα levels were inhibited in KLE^TFEB−OV^ cells and ECC-1^TFEB−OV^ after XCT790 treatment. The OCR was no significant increase in KLE^TFEB−OV+XCT790^ cells and ECC-1^TFEB−OV+XCT790^ cells(p > 0.05; Fig. 4B).

The degree of saturation of fatty acyl moieties of membrane phospholipids determines the biophysical properties of cell membranes, such as their fluidity. LPCAT1 and LPCAT3 are the key phospholipid remodeling enzymes that regulate the degree of saturation of fatty acyl moieties in the membrane [16]. Thus, we tested the FA desaturase proteins LPCAT1 and LPCAT3. LPCAT1/3 were increased in KLE^ERRα−OV^ compared with the controls. In contrast, both LPCAT1 and LPCAT3 decreased in KLE^ERRα−KD^ compared with the controls (p < 0.05). Similarly, MMP2 and Cortactin were altered with ERRα which detected to evaluate membrane fluidity (p < 0.05; Fig. 4C). A similarly trend of LPCAT1/3, MMP2 and Cortactin was observed in ECC-1 cells. Furthermore, LPCAT1/3, MMP2 and Cortactin were inhibited apparently when we treated KLE and ECC-1 with XCT790. However, these proteins could not be reversed by XCT790 when TFEB was over-expressed (p < 0.05; Fig. 4D). To observe the generation of pseudopod, we found there were more filopodia in KLE^TFEB−OV^ and KLE^ERRα−OV^ than KLE cells, while fewer thin filopodia were scanned in KLE^ERRα−KD^ and KLE with XCT790 treatment by SCE. Moreover, TFEB over-expressed annulled the inhibitory effect of XCT790 to keep the filopodia and pseudopod. Consistent with KLE cell pseudopodia, TFEB-ERRα promote the filopodia and the outcome of XCT790 treatment could be withdrew as TFEB was up-regulated. (Fig. 4E) These data suggested that TFEB could induce mitochondrial stress and phospholipid remodeling to elevate the fluidity of the cell membrane by upregulating ERRα in KLE and ECC-1 cells.

### TFEB promotes EC migration depending on ERRα via EMT signaling

**Fig. 5:**
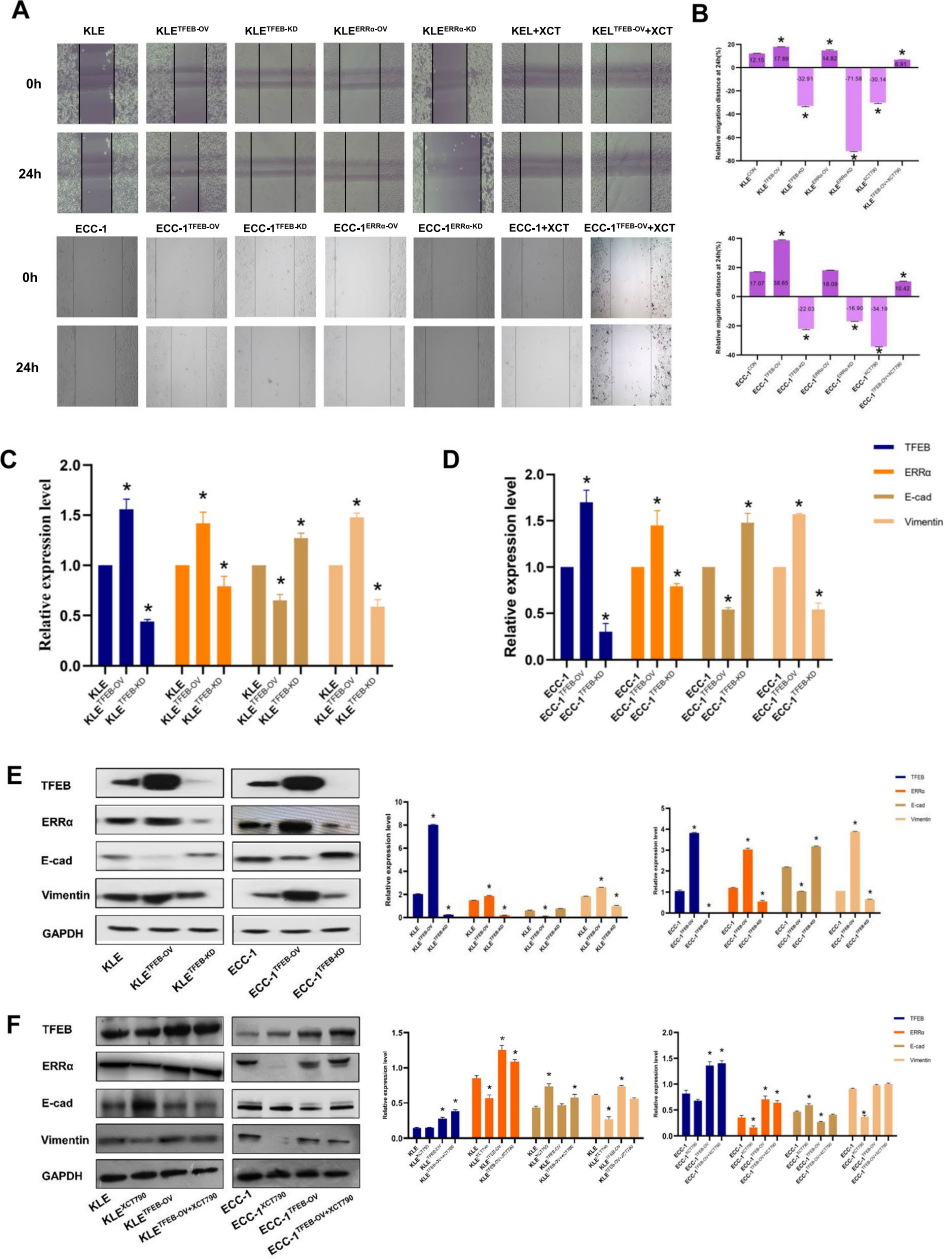
TFEB promotes EC migration depending on ERRα via EMT signaling (**A-B**) Wound healing of TFEB-ERRα regulation in KLE and ECC-1 cells. (**C-D**) TFEB regulates ERRα, E-cadherin, and vimentin expression in EC cells is analyzed using RT-qPCR. (**E–F**) The effect of XCT790 treatment for 24 h on TFEB over-expressing in EC cells. *, p < 0.05. Statistical tests: Student’ s *t*-test.

Although the role of ERRα in promoting EC invasion and metastasis has been established [9], its underlying mechanisms are far from elucidated. The migration ability of KLE and ECC-1 cells was remarkably changed after regulation of the expression of TFEB or ERRα through a lentivirus-mediated strategy. Compared to the controls, the scratched spaces were up to 47.2% in KLE^TFEB−OV^, 21.9% in KLE^ERRα−OV^ at 24 h and up to 126.4% in ECC-1^TFEB−OV^, respectively (both p < 0.05; Fig. 5A-B). The scratched spaces of ECC-1^ERRα−OV^ at 24 h also increased slightly, but there was no significant difference from their controls (p > 0.05). The wounded spaces were nearly two-fold decreased at 24 h in both KLE^TFEB−KD^ and KLE^ERRα−KD^ cells compared with their controls (p < 0.001). A similar trend was observed in ECC-1^TFEB−KD^ and ECC-1^ERRα−KD^. To further confirm the effects of TFEB on ERRα-mediated cell migration, ERRα levels were inhibited in KLE^TFEB−OV^ cells and ECC-1^TFEB−OV^ cells after XCT790 treatment. The scratched space was not significantly increased from 0 to 24 h in KLE^TFEB−OV+XCT790^ cells or ECC-1^TFEB−OV+XCT790^ cells. Meanwhile, the enhanced migration abilities of KLE^TFEB−OV^ and ECC-1^TFEB−OV^ cells were partially compromised after XCT790 treatment (p < 0.05; Fig. 5A-B). Moreover, the RT-qPCR results showed that downregulation of TFEB reduced the expression of ERRα and vimentin and increased the expression of E-cadherin in ECC-1 and KLE cells. In contrast, TFEB overexpression enhanced the expression of ERRα and vimentin and decreased the expression of E-cadherin (p < 0.05, Fig. 5C-D). These data are similar to the findings observed in Western blot experiments (p < 0.05, Fig. 5E). Importantly, there was no significant change in ERRα, vimentin or E-cadherin expression when KLE^TFEB−OV^ and ECC-1^TFEB−OV^ cells were treated with 10 µM XCT790 (p > 0.05, Fig. 5F). This demonstrated that TFEB could regulate cell migration in an ERRα-dependent manner via the EMT signaling pathway.

### High expression of TFEB-ERRα is associated with dyslipidemia and metastasis in EC patients

**Fig. 6:**
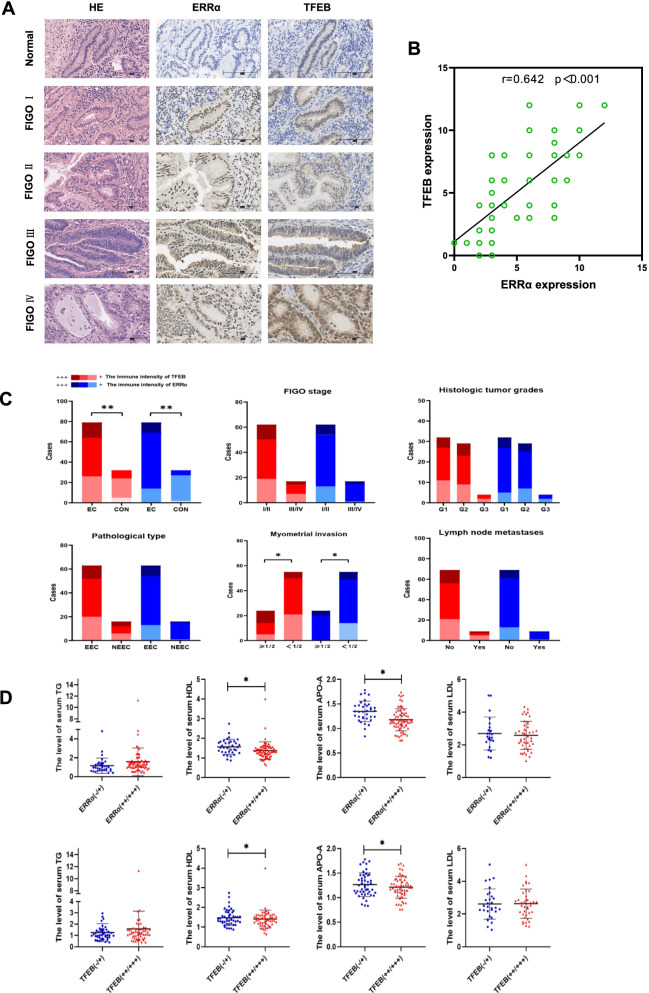
High expression of TFEB-ERRα is associated with dyslipidemia and metastasis in EC patients The immunohistochemical expression of several proteins and their correlations are shown. **(A)** The immunohistochemical expression of TFEB and ERRα in the normal endometrium (n = 32) and EC at different FIGO stages (n = 79) (magnification: × 400). (**B)** The correlation between TFEB and ERRα expression is shown in tissues. (**C)** Analysis of TFEB-ERRα and clinicopathological features. (**D)** The differences level of serum TG, HDL, APOA and LDL in groups with different expression levels of TFEB. *, p < 0.05. Statistical tests: Student’s *t*-test (D), pearson’s rank correlation analysis (C). MI: Myometrial Invasion; LNM: Lymph Node Metastases; TG: Triglyceride; HDL: High-Density Lipoprotein; APOA: Apolipoprotein A; LDL: Low-Density Lipoprotein.

To verify the results obtained above, IHC was performed on an EC tissue microarray (TMA), which included 79 EC specimens and 32 normal endometrium specimens. Positive immunoreactivity for TFEB was detected in the nuclei of both carcinoma cells and normal endometrial gland cells. Significantly higher immunoreactivity was observed in EC tissue than in normal endometrial tissue (p < 0.001; Fig. 6A & Table 1). Similarly, ERRα could also be detected in 79 EC tissue samples with a higher immunoreactivity than it detected in 32 normal endometrial tissues (p < 0.001; Fig. 6A & Table 1). There was no significant difference in TFEB or ERRα expression among EC patients with different International Federation International of Gynecology and Obstetrics (FIGO) stages, histologic tumor grades, pathological types or lymph node metastasis (LNM) conditions (p > 0.05; Fig. 6C). However, significant differences were detected between the ≤ 1/2 and > 1/2 myometrial invasion (MI) groups for both TFEB and ERRα expression in EC patients (p < 0.05; Fig. 6C). Moreover, a positive correlation between TFEB and ERRα immunoreactivity was found based on Pearson’s rank correlation analysis (r = 0.642, p < 0.001, Fig. 6B).

**Table 1:** The level of serum lipid, ERRα and TFEB in tissue microarray of EC patients and controls

**Parameter **(N=111)	**TG**	** p**	**CHOL**	** p**	**HDL**	** p**	**APO-A**	** p**	**LDL**	** p**	**APO-B**	** p**	**TFEB **				**ERRα**			
													-/+	++	+++	p	-/+	++	+++	p
Normal (n=32)	1.027±0.882	**0.032**	4.623±0.853	0.649	1.599±0.412	**0.041**	1.331±0.229	**0.026**	2.709±0.830	0.568	0.834±0.229	0.157	24	8	0	**<0.001**	27	5	0	**<0.001**
EC (n=79)	1.549±1.369		4.723±1.032		1.394±0.428		1.207±0.226		2.578±0.929		0.924±0.338		26	38	15		14	55	10	
Stage I-II (n=62)	1.556±1.538	0.859	4.703±1.133	0.192	1.388±0.470	0.980	1.191±0.224	0.691	2.693±0.985	0.178	0.935±0.381	0.785	19	31	12	0.710	13	41	8	0.328
Stage III-IV (n=17)	1.622±1.019		5.055±0.719		1.386±0.234		1.218±0.210		3.148±0.636		0.958±0.236		7	7	3		1	14	2	
MI <50% (n=55)	1.597±1.531	0.589	4.742±1.064	0.806	1.388±0.463	0.827	1.196±0.219	0.550	2.593±0.979	0.880	0.936±0.362	0.618	21	29	5	**0.003**	14	35	6	**0.024**
MI ≥50% (n=24)	1.445±0.933		4.680±0.979		1.408±0.350		1.231±0.244		2.553±0.869		0.898±0.282		5	9	10		0	20	4	
EEC (n=63)	1.515±1.397	0.592	4.790±1.056	0.184	1.424±0.452	0.120	1.218±0.225	0.390	2.706±0.976	**0.021**	0.950±0.353	0.100	20	32	11	0.613	13	41	9	0.216
NEEC (n=16)	1.711±1.256		4.412±0.887		1.274±0.301		1.162±0.232		2.133±0.578		0.819±0.254		6	6	4		1	14	1	
LNM (n=9)	1.605±1.447	0.316	4.724±1.059	0.729	1.367±0.421	**0.046**	1.193±0.223	0.151	2.640±0.946	0.092	0.925±0.351	0.984	5	2	2	0.232	1	7	1	0.842
No-LNM (n=70)	1.413±0.380		4.836±0.812		1.637±0.439		1.326±0.242		1.980±0.666		0.923±0.253		21	36	13		13	48	9	

*Abbreviations: APO-A* Apolipoprotein A, *APO-B* Apolipoprotein B, *CHOL* Cholesterol, *ERRα* Estrogen-Related Receptor α, *EC* Endometrial Cancer, *EEC* Endometrioid Endometrial Cancer, *HDL* High-Density Lipoprotein, *LDL* Low-Density Lipoprotein, *LNM* Lymph Node Metastasis, *MI* Myometrial Invasion, *NEEC* Non-Endometrioid Endometrial Cancer, *TG* total Triglyceride, *TFEB* Transcription Factor EB. p < 0.05 suggests significantly different

Interestingly, in a study on the same population for serum lipids, the serum total triglyceride (TG) level was significantly higher, while the high-density lipoprotein (HDL) and apolipoprotein A (APO-A) levels were significantly lower in EC patients than in health people (P < 0.05, Table 1). In addition, the low-density lipoprotein (LDL) level was obviously higher in patients with endometrioid endometrial cancer (EEC) than in patients with non-endometrioid endometrial cancer (NEEC). Importantly, both HDL and APO-A levels were decreased significantly in patients with LNM (p < 0.05, Table 1). Next, we compared the differences in serum lipids in populations with different TFEB/ERRα expression levels. Not unexpectedly, serum HDL and APO-A levels were much lower in the patients with high expression of TFEB and ERRα (+ + / +  + +) than in those with low TFEB and ERRα expression (-/ +) (p < 0.05; Fig. 6D). Given from  the evidence above, elevated TFEB-ERRα is involved in EC invasion and metastasis and is related to decreases in serum HDL and APO-A levels.

### Accumulation of UFA-containing GPs induced by ERRα is required for EC progression

**Fig. 7:**
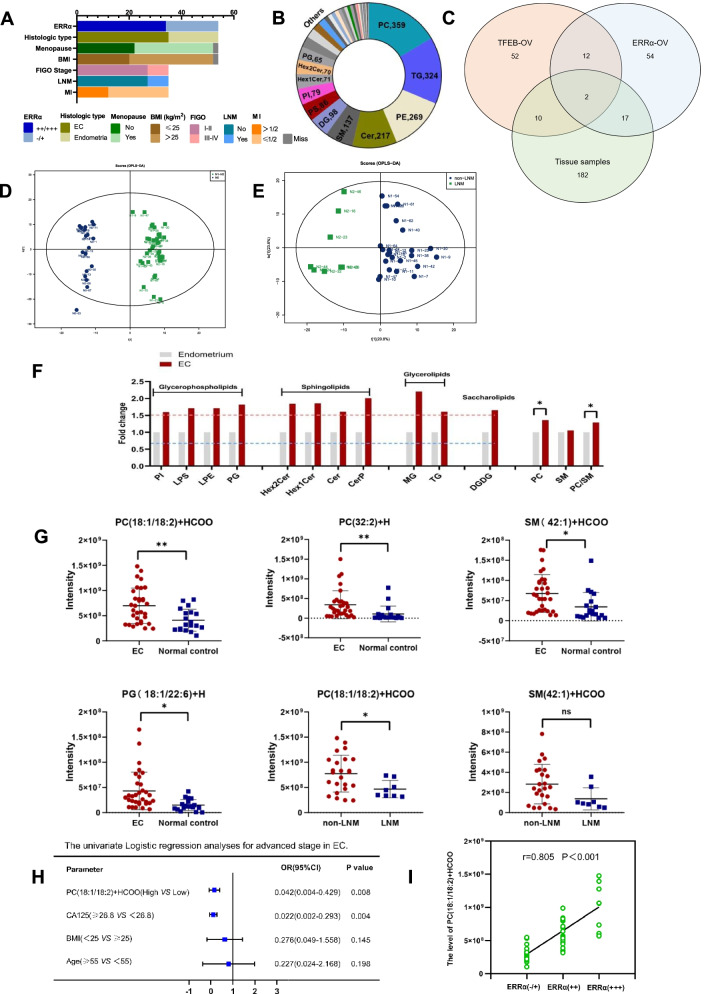
Accumulation of UFA-containing GPs induced by ERRα is required for EC progression **(A)** The baseline characteristic including BMI, menopause, FIGO stage, histologic type, MI, LNM and ERRα expression of patients (EC = 35 vs controls = 19) tissues for lipidomic. (**B)** Different classes of lipids were tested in 35 EC tissues and 19 control tissues. (**C)** Venn diagram showing the distribution of TFEB-ERRα axis related lipids and ERRα-dependent lipids in clinical samples. (**D**)Systematic lipidomic changes between EC and control tissues assessed by OPLS-DA. (**E**) Systematic lipidomic changes between LNM and non-LNM tissues assessed by OPLS-DA. (**F**) Significant lipid species in EC and normal control tissues and its PC/SM evaluation. (G) Analysis of TFEB-ERRα associated lipids, ERRα-dependent lipids and EC as well as LNM. (**H**) The univariate binary logistic regression analyses of Age, BMI, CA125, PC (18:1/18:2) + HCOO and PC (32:2) + H. (**I**) The correlation analysis of PC (18:1/18:2) + HCOO and ERRα. *, p < 0.05. Statistical tests: Student’ s *t*-test(E–F), Logistic regression(G), Pearson’s rank correlation analysis(H). BMI: Body Mass Index.

The species of lipids obtained from 32 cases of EC tissues and 19 cases normal endometrial tissues (3 cases were disqualified) were similar to the cells, which included 359 PCs, 324 TAGs, 269 PEs and other lipid species (Fig. 7A-B). Venn showed that 2 lipids were found in three groups, including PG (18:1/22:6) + H, PC (36:3) + H and 19 lipids were detected both in KLE^ERRα−OV^ and clinical tissues (Fig. 7C). Systematic lipidomic changes occurring between EC patients and normal controls were assessed by OPLS-DA. There was obvious heterogeneity between the populations, with R^2^Y = 0.963 and Q^2^ = 0.510 (Fig. 7D). Fair discrimination was also found between patients with LNM (n = 8) and those without LNM (n = 24). (R^2^Y = 0.789 and Q^2^ = 0.574, Fig. 7E). Consistent with the results from in vitro research in cells, DGDG was observed to increase with a fold change (FC) > 1.5. In addition, PGs, including phosphatidylinositol (PI), lysophosphatidylserine (LPS), lysophosphatidylethanolamine (LPE) and PG, sphingolipids (SLs), including Hex2Cer, Hex1Cer, Cer and ceramide phosphate (CerP), and GLs, including monoglyceride (MG) and TG, were also increased in EC tissue. Although SM was elevated indistinctively, PC/SM was significantly increased in EC tissue, which was used to evaluate cell membrane fluidity (p < 0.05, Fig. 7F). Moreover, UFA-containing GPs, such as PC (18:1/18:2) + HCOO, PC (32:2) + H, PG (18:1/22:6) + H and SM (42:1) + HCOO, were obviously increased in EC tissue compared to normal endometrium (FC > 1.5, VIP > 1 and p < 0.05; Fig. 6F). However, compared to patients without LNM, PC (18:1/18:2) + HCOO was much higher in the patients with LNM (p < 0.05; Fig. 7G). We performed ROC curve analysis and obtained the cutoff values. The results showed that PC (18:1/18:2) + HCOO and CA125 have good diagnostic value in patients with advanced-stage EC (p < 0.05; Supplement Figure 2). Moreover, PC (18:1/18:2) + HCOO was found to be an independent risk factor for EC patients in advanced stage by univariate binary logistic regression analyses (p < 0.05, Fig. 7H).

Furthermore, we compared the differences in serum lipids in populations with different PC (18:1/18:2) + HCOO expression levels. The results showed that the serum HDL level was much lower in the patients with higher expression of PC (18:1/18:2) + HCOO than in those with lower PC (18:1/18:2) + HCOO (p < 0.05, Table 2). Moreover, a positive correlation between PC (18:1/18:2) + HCOO and ERRα immunoreactivity was found based on Pearson’s rank correlation analysis (r = 0.805, p < 0.001, Fig. 7I). These results suggested that increased PC (18:1/18:2) + HCOO induced by ERRα was a novel biomarker of EC progression, which was probably related to the decrease in serum HDL.

**Table 2:** Associations of PC(18:1/18:2)+HCOO with serum lipid

Parameter	PC(18:1/18:2)+HCOO<cutoff value (n=23)	PC(18:1/18:2)+HCOO≥cutoff value (n=28)	p
TG(mmol/L)	1.533±0.650	2.911±9.981	0.136
CHOL(mmol/L)	5.379±1.062	5.329±1.258	0.904
**HDL**(mmol/L)	1.416±0.345	1.190±0.313	**0.028**
APO-A(g/L)	1.083±0.130	1.057±0.182	0.645
LDL(mmol/L)	3.367±0.958	2.960±0.757	0.221
APO-B(g/L)	1.011±0.261	0.961±0.200	0.573

*Abbreviations:* *APO-A* Apolipoprotein A, *APO-B* Apolipoprotein B, *CHOL* Cholesterol, *HDL* High-Density Lipoprotein, *LDL* Low-Density Lipoprotein, *PC* Phosphatidylcholine, *TG* total Triglyceride, Cut-off value=521540036.5. p < 0.05 suggests significantly different

In conclusion, the TFEB-ERRα axis promotes UFA-containing GP accumulation to induce lipid reprogramming hallmarked by mitochondrial stress, which contributes to the invasion and metastasis of EC (Supplement Figure 3). Importantly, UFA-containing PC, SM and PG were the significantly altered lipids found in EC cells and tissues, among which, PG (18:1/22:6) + H induced by TFEB-ERRα axis was involved in tumorigenesis and PC (18:1/18:2) + HCOO was the ERRα-dependent lipid to mediate EC metastasis.

## Discussion

EC is one of the cancers most related to metabolic disorders, and patients present with hyperlipidemia, hyperglycemia, hypertension and other clinical symptoms [21]. Guo et al. suggested that metformin significantly reversed obesity-driven lipid and protein biosynthesis upregulation in an obese LKB1^fl/fl^ p53^fl/fl^ mouse model of EC [22]. Recently, an increasing number of studies have confirmed that ERRα is a key regulator of metabolism in obesity-related tumors, such as breast cancer [23], prostate cancer [24], and EC [25]. Moreover, ERRα-mediated signaling pathways have recently emerged as key factors in the regulation of cancer lipid metabolism. In our previous work, the translational factor activity of TFEB was affected by the downregulation of ERRα expression in EC according to a high-throughput DNA/protein assay [25], which suggested that TFEB should interact with ERRα and be involved in EC lipid reprogramming and progression, which triggered our interest. TFEB downregulation or deficiency can obviously affect the cellular phenotype in a physiologically relevant manner in settings including atherosclerosis [26], nonalcoholic fatty liver disease [27], cancer [28] and neurodegeneration [29]. Furthermore, TFEB is activated by starvation or caloric restriction and plays roles in lipid catabolism and lysosomal biogenesis [14]. Therefore, we started with bioinformatics analysis of the TCGA data. In agreement with our hypothesis, the results confirmed that both TFEB and ERRα are strongly associated with a poor prognosis in EC, as reflected by their associations with a high FIGO stage and a shortened survival time. Moreover, the expression of TFEB was first found to be positively correlated with the expression of ERRα in the TCGA data of 543 EC cases. The bioinformatics analysis result was further verified by our clinical data from our TMA, in which TFEB and ERRα showed a strong correlation and both were related to the MI of EC. However, the exact interaction mechanism between TFEB and ERRα has not yet been described clearly. In 2019, TFEB was reported to drive PGC-1α expression in adipocytes to protect against diet-induced metabolic dysfunction, while PGC-1α is one of the most important coactivators of ERRα [30]. We further confirmed that TFEB can bind to the promoter region of ERRα and regulate the expression and function of ERRα in vitro by ChIP assay and luciferase assay.

Previously, we reported that high expression of ERRα is associated with cancer cell metastasis and invasion [11]. To our knowledge, this is the first report indicating that TFEB participates in the invasion of EC cells by EMT signaling. Interestingly, the patient's clinical lipid profile indicated that serum HDL and APO-A were negatively correlated with TFEB and ERRα. Lower serum HDL and APO-A levels were associated with LNM in EC patients. As we know, PC was the major phospholipids of HDL [31]. Importantly, we found a significant decrease in HDL in the patients with higher PC (18:1/18:2) + HCOO, which indicated that PC remodeling probably influenced the serum HDL level of EC patients. Together, TFEB/ERRα was an early predictor of MI, while lower HDL/APO-A and higher PC (18:1/18:2) + HCOO were risk factors for LNM in advanced EC. In brief, TFEB/ERRα regulates EC patients’ lipid metabolism and is involved in EC invasion and metastasis.

Moreover, our in vitro and in vivo lipidomics experiments first investigated ERRα as a downstream signal by which TFEB promotes UFA-GP accumulation during EC progression. Guo found dramatic increases in lipid biosynthesis and lipid peroxidation in a genetically engineered mouse model of endometrioid adenocarcinoma [22], suggesting that lipidomic changes or reprogramming are significant in EC. Previous studies have confirmed that downregulation of ERRα provides a potential therapeutic strategy and inhibits cellular metastasis and invasion in EC [11, 25]. However, the mechanism by which TFEB-ERRα regulates FA and GP synthesis was unexplored prior to our study. After overexpressing TFEB and ERRα, the top three categories of lipids obtained were GPs, SPs and GLs. Among them, PC (36:3) + H, PC (35:1) + H and PG (18:1/22:6) + H were found to be the significantly lipids and PG (18:1/22:6) + H was the lipid positively related to TFEB-ERRα overexpression, which was also elevated in cancerous tissues. This evidence indicated an unexpected role of TFEB-ERRα in FA unsaturated and phospholipid remodeling pathways that are controlled by rate-limiting metabolic enzymes, including LPCAT1 and LPCAT3 [32, 33]. The impacts of UFA-containing GP accumulation on membrane fluidity have been proposed as secondary effects linked to desaturation induced by LPCAT1 and LPCAT3. The sensitivity of lipids to oxidative stress depends on their fatty acid moiety. Double bonds in MUFAs and PUFAs show a prevalent *cis* conformation, which produces bends and limits their rigid packing [34]. Since double bonds make fatty acyl chains more susceptible to oxidative stress, increased UFA-GPs promote membrane fluidity when ATP is elevated in this study. Commonly cancer cells possess different and complementary metabolic profiles, microenvironments and adopting behaviors to generate more ATPs to fulfill the requirement of high energy, which is further utilized in the production of proteins and other essential processes required for cell survival, growth, and proliferation. Mitochondria are partially autonomous organelles that depend on the import of certain proteins and lipids to maintain cell survival and membrane formation [35]. CL and/or PG are considered mitochondria-specific phospholipids [36]. PG (18:1/22:6) was found to be increased in MYC-induced T cell acute lymphoblastic leukemia, renal cell carcinoma, hepatocellular carcinoma, and lung carcinoma [37]. Our results showed that PG (18:1/22:6) + H was much higher in EC patients, which demonstrated the important role of mitochondria in maintaining membrane homeostasis. The synthesis of mitochondrial ATP plays a key role in inducing membrane curvature to establish cristae in eukaryotes [38]. The effect of mitochondrial stress was considered as the hallmark of membrane remodeling, which demonstrated herein that TFEB-ERRα enhanced membrane fluidity by stimulating mitochondria to prepare for invasion and metastasis. However, mitochondria lacks PC synthesizing enzymes, and this lipid has to be imported from other organelles, such as the endoplasmic reticulum (ER). The prevailing view is that a significant pool of cellular PC can also be made de novo from PS in a pathway that originates in the ER and passes into and out of the mitochondrion [39]. The genes related to FA metabolism, such as *acc, fasn* and *acadm,* increased with ERRα, which suggested that ERRα mobilized β-oxidation and de novo lipogenesis to facilitate lipid reprogramming. In addition, increased PC (18:1/18:2) + HCOO has been shown to be a good predictor for prostate cancer [40]. In line with these findings, we found that PC (18:1/18:2) + HCOO was not only related to EC but also associated with EC metastasis, which showed a potential role of a tumor marker.

In summary, we found that the TFEB-ERRα signaling pathway regulates the invasion and metastasis of endometrial cancer cells through the EMT pathway and cell membrane fluidity. This regulation depends on ERRα to participate in the metabolism of lipids and cellular membrane remodeling. TFEB-ERRα enhances UFA-PCs, PG (18:1/22:6) + H and PC/SM in EC patients to promote cellular fluidity and results in invasion and metastasis. Furthermore, PC (18:1/18:2) + HCOO is the ERRα-dependent potential lipid to mediate EC metastasis. This also explains why ERRα, as a key factor in energy metabolism, is a poor prognostic factor for EC.

## Supplementary Information

Additional file 1: **Figure S1**. The flow chart of study participants. Abbreviation: APO-A, a polipoprotein A; APO-B, a polipoprotein B; BMI, Body Mass Index; CA125, Cancer antigen 125; CHOL, Cholesterol; Con, Control; EC, Endometrial cancer; ERRα, Estrogen-Related Receptor α; FIGO, Federation International of Gynecology and Obstetrics; HDL, High-Density Lipoprotein; IHC, Immunohistochemistry; IRS,Immunoreactive Score; LDL, Low-Density Lipoprotein; LNM, Lymph Node Metastasis; LC/MS, Liquid Chromatography Mass Spectrometry; PC, Phosphatidylcholine; PE, Phosphatidylethanolamine; TAG, Triacylglycerol; TG, Total Triglyceride; TFEB, transcription factor EB. **Figure S2**. The ROC cruve of PC (18:1/18:2) +HCOO, PC (32:2) +H, HDL and CA125. **Figure S3**. Hypothesis diagram of lipid reprogramming in EC cells modulated by TFEB-ERRα axis. **Table S1**. The overlapped lipids between TFEB and ERRα over-expressing. **Table S2**. Significant proteins derived from omics analysis. 

## Data Availability

The datasets analyzed during the current study are available from the corresponding author on reasonable request.

## Abbreviations

ACADM: Medium-chain Acyl-coenzyme A Dehydrogenase
ACC: Acetyl-Co A Carboxylas
APO-A: Apolipoprotein A
ATCC: American Type Culture Collection
ATP: Adenosine Triphosphate
BMI: Body Mass Index
Cer: Ceramide
CerP: Ceramides Phosphate
ChIP-qPCR: Chromatin Immunoprecipitation Quantitative Polymerase Chain Reaction
CHOL: Cholesterol
CL: Cardiolipin
CNV: Copy Number Variation
DAVID: The Database for Annotation, Visualization and Integrated Discovery
DGDG: Digalactosyldiacylglycerol
DMEM: Dulbecco's Modification of Eagle's Medium Dulbecco
DMSO: Dimethyl Sulfoxide
EC: Endometrial Cancer
EEC: Endometrioid Endometrial Cancer
EMT: Epithelial-Mesenchymal Transformation
ERα: Estrogen receptor α
ERRα/ NR3B1/ESRRA: Estrogen-Related Receptor α
FASN: Fatty Acid Synthase
FBS: Fetal Bovine Serum
FC: Fold Change
FCCP: Carbonyl cyanide 4-(trifluoromethoxy)phenylhydrazone
FIGO: Federation International of Gynecology and Obstetrics
GL: Glycerolipid
GM2: Gangliosides2
GP: Glycerophospholipid
GSEA: Gene Set Enrichment Analysis
HDL: High-Density Lipoprotein
HPLC: High Pressure Liquid Chromatography
IHC: Immunohistochemistry
IRS: Immunoreactive Score
KEGG: Kyoto Encyclopedia of Genes and Genomes
LC/MS: Liquid Chromatography Mass Spectrometry
LDL: Low-Density Lipoprotein
LNM: Lymph Node Metastasis
LPCAT: Lysophosphatidylcholine Acyltransferase
LPS: Lysophosphatidylserine
LPE: Lysophosphatidylethanolamine
MG: Monoglyceride
MI: Myometrial Invasion
MMP2: Matrix Metalloproteinase 2
NEEC: Non-Endometrioid Endometrial Cancer
OCR: Oxygen consumption rate
OPLS-DA: Orthogonal Partial Least Squares-Discriminant Analysis
OS: Overall Survival
PBS: Phosphate Buffered Solution
PC: Phosphatidylcholine
PE: Phosphatidylethanolamine
PG: Phosphatidylglycerol
PI: Phosphatidylinositol
PGC-1α: Peroxisome Proliferator-Activated Receptor Coactivator-1α
PPAR: Peroxisome Proliferator-Activated Receptor
PS: Phosphatidylserine
RT-qPCR: Reverse Transcription quantitative Polymerase Chain Reaction
ROC: Receiver Operating Characteristic
SFA: Saturated Fatty Acid
siRNA: Small Interfering RNA
SLs: Sphingolipids
SM: Sphingomyelin
SEM: Scanning Electron Microscope
TAG: Triacylglycerol
TCGA: The Cancer Genome Atlas
TFEB: Transcription Factor EB
TG: total Triglyceride
TMA: Tissue Microarray
TMT: Tandem Mass Tags
UCEC: Uterine Corpus Endometrial Carcinoma
UCSC: University of California, Santa Cruz
UFA: Unsaturated Fatty Acid
WB: Western Blotting
