# Supplementary material for: Lipid reprogramming induced by the TFEB-ERRα axis enhanced membrane fluidity to promote EC progression
Source: J Exp Clin Cancer Res. 2022 Jan 19;41:28. doi: 10.1186/s13046-021-02211-2 (PMC8767755; doi:10.1186/s13046-021-02211-2)
Supplement: Supplementary file 1 — Additional file 1: Figure S1. The flow chart of study participants. Abbreviation: APO-A, a polipoprotein A; APO-B, a polipoprotein B; BMI, Body Mass Index; CA125, Cancer antigen 125; CHOL, Cholesterol; Con, Control; EC, Endometrial cancer; ERRα, Estrogen-Related Receptor α; FIGO, Federation International of Gynecology and Obstetrics; HDL, High-Density Lipoprotein; IHC, Immunohistochemistry; IRS,Immunoreactive Score; LDL, Low-Density Lipoprotein; LNM, Lymph Node Metastasis; LC/MS, Liquid Chromatography Mass Spectrometry; PC, Phosphatidylcholine; PE, Phosphatidylethanolamine; TAG, Triacylglycerol; TG, Total Triglyceride; TFEB, transcription factor EB. Figure S2. The ROC cruve of PC (18:1/18:2) +HCOO, PC (32:2) +H, HDL and CA125. Figure S3. Hypothesis diagram of lipid reprogramming in EC cells modulated by TFEB-ERRα axis. Table S1. The overlapped lipids between TFEB and ERRα over-expressing. Table S2. Significant proteins derived from omics analysis. [file 13046_2021_2211_MOESM1_ESM.zip › supplement-table-1-lipidomic-TOV-EOV-new.docx]

Supplement table 1

The overlapped lipids between TFEB and ERRα over-expressing

| **Lipids** | **TFEB-OV**  **(Fold Change)** | **ERRα-OV**  **(Fold Change)** |
| --- | --- | --- |
| CL(18:1/16:0/16:0/18:1)+H | Down (0.258807) | Up (1.540929) |
| CL(20:4/16:0/16:0/18:1)+H | Down (0.357985) | Up (1.540929) |
| CL(18:1/16:0/18:1/14:0)+H | Down (0.40366) | Up (1.508683) |
| PC(37:5)+H | NS (0.682139) | NS (0.720664) |
| PC(33:0)+H | Down (0.57888) | NS (0.781515) |
| **PC(36:3)+H** | **Down (0.562871)** | **Down (0.648363)** |
| PC(15:0/16:0)+HCOO | Down (0.500586) | NS (0.732324) |
| **PC(35:1)+H** | **Down (0.569485)** | **Down (0.605667)** |
| PE(18:1/22:1)+H | Down (0.655292) | NS (0.814202) |
| PE(18:0/22:5)+H | NS (0.720781) | NS (0.681302) |
| PC(16:0/17:0)+HCOO | Down (0.531906) | NS (0.75081) |
| PG(16:0/16:1)+H | Down (0.583468) | NS (1.316118) |
| **PG(18:1/22:6)+H** | **Up (1.540214)** | **Up (2.157884)** |
| SM(d40:1)+H | NS (0.758915) | NS (0.755688) |

Overlapped lipids: the lipids with fold change (FC)<0.67 or FC>1.5 and p<0.05. CL: cardiolipin; PC: phosphatidylcholine; PE: phosphatidylethanolamine; PG: phosphatidylglycerol; SM: sphingomyelin; NS: no significant.
