# Supplementary figures and images for: Lipid reprogramming induced by the TFEB-ERRα axis enhanced membrane fluidity to promote EC progression
Source: J Exp Clin Cancer Res. 2022 Jan 19;41:28. doi: 10.1186/s13046-021-02211-2 (PMC8767755; doi:10.1186/s13046-021-02211-2)

## Slide 1
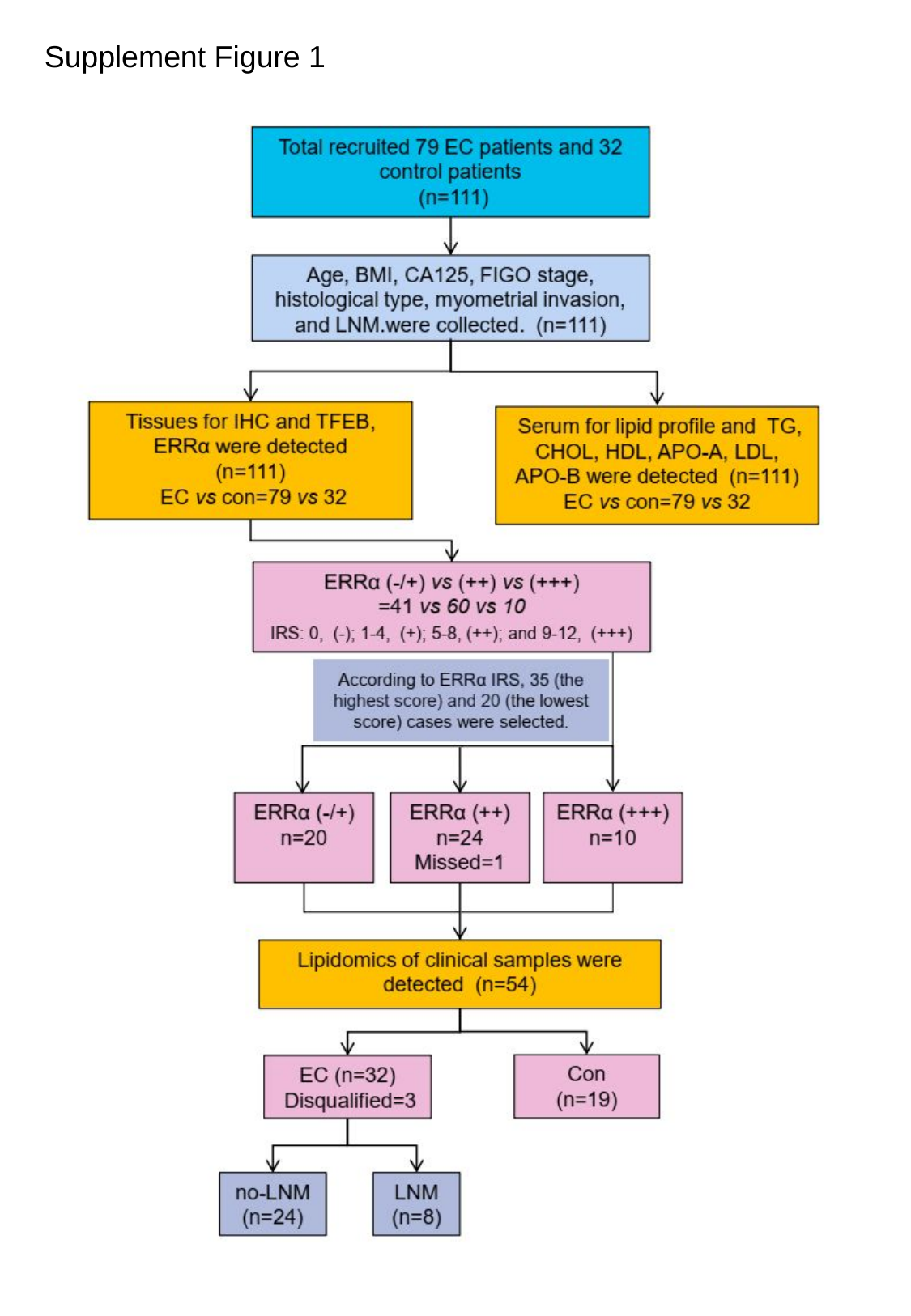

Supplement Figure 1

## Slide 2
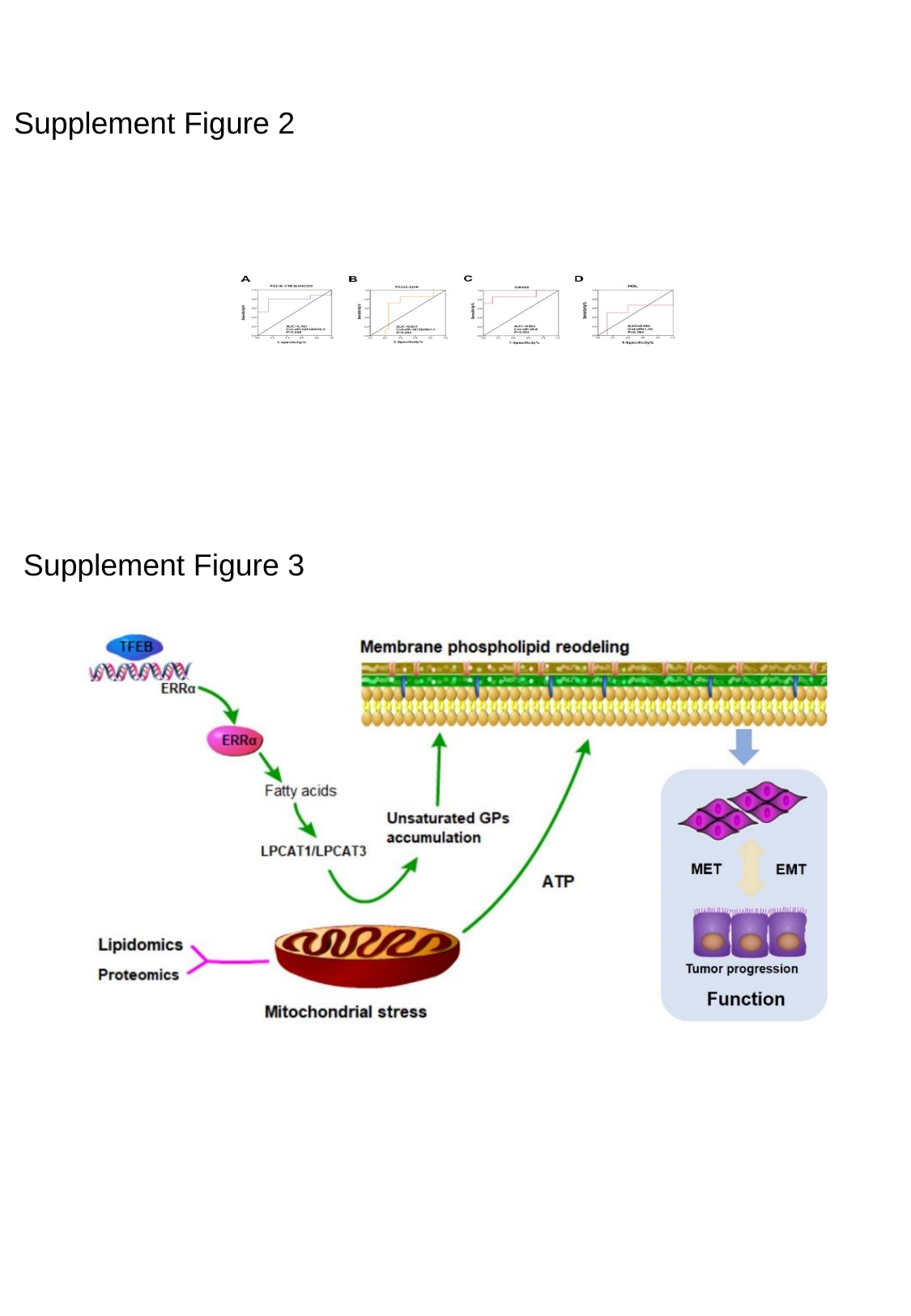

Supplement Figure 2
Supplement Figure 3

Supplement: Supplementary file 1 — Additional file 1: Figure S1. The flow chart of study participants. Abbreviation: APO-A, a polipoprotein A; APO-B, a polipoprotein B; BMI, Body Mass Index; CA125, Cancer antigen 125; CHOL, Cholesterol; Con, Control; EC, Endometrial cancer; ERRα, Estrogen-Related Receptor α; FIGO, Federation International of Gynecology and Obstetrics; HDL, High-Density Lipoprotein; IHC, Immunohistochemistry; IRS,Immunoreactive Score; LDL, Low-Density Lipoprotein; LNM, Lymph Node Metastasis; LC/MS, Liquid Chromatography Mass Spectrometry; PC, Phosphatidylcholine; PE, Phosphatidylethanolamine; TAG, Triacylglycerol; TG, Total Triglyceride; TFEB, transcription factor EB. Figure S2. The ROC cruve of PC (18:1/18:2) +HCOO, PC (32:2) +H, HDL and CA125. Figure S3. Hypothesis diagram of lipid reprogramming in EC cells modulated by TFEB-ERRα axis. Table S1. The overlapped lipids between TFEB and ERRα over-expressing. Table S2. Significant proteins derived from omics analysis. [file 13046_2021_2211_MOESM1_ESM.zip › Figure-46.pptx]
